# Management of stored grain pest with special reference to *Callosobruchus maculatus*, a major pest of cowpea: A review

**DOI:** 10.1016/j.heliyon.2021.e08703

**Published:** 2022-01-01

**Authors:** Kalpna, Younis Ahmad Hajam, Rajesh Kumar

**Affiliations:** a Division Zoology, Department of Biosciences, Career Point University, Hamirpur, Himachal Pradesh, 176041, India; b Department of Biosciences, Himachal Pradesh University, Shimla, Himachal Pradesh, 171005, India

**Keywords:** Pest, Bruchids, *Callosobruchus*, Pulses, Management

## Abstract

Bruchids are most pernicious pest of stored grain pulses, especially in the tropical and subtropical areas. They penetrate into the fully grown matured pods, grains in fields and also during post-harvest storage. Among bruchids, *Callosobruchus maculatus* is the prominent pest having ubiquitous distribution. Chemical/synthetic insecticides provides adequate control against the *C. maculatus* on the pulses. However, the use of synthetic insecticides induces adverse health outcomes in agricultural workers and many causes various diseases such as cancers, genomic damage, oxidative stress, neurological disorders and respiratory, metabolic and thyroid effects. Therefore, alternative effective, safe and sustainable pest control, integration of different compatible methods should be taken into considerations. One of the possible managements might be use of traditional as well modern pest management practices. Traditional techniques include sealed containers, inert materials, harvesting time, alternate host, intercropping, storing un-threshed pulses, cleanliness, vegetable oil etc. Modern techniques such as temperature, freezing and heating, radiation treatments, resistance varieties, natural control, botanical extracts, chemical and microbial, transgenic approach, cold plasma treatments etc. thus integrated pest management might be alternative approach to combat the effect of pest. Therefore, present review aims to considers various measures for the handling of bruchids with special reference to *Callosobruchus maculatus* and integrated molecular inventions to decrease bruchids populations and enhance pulse productivity in pulses.

## Graphical abstract

**Figure:**
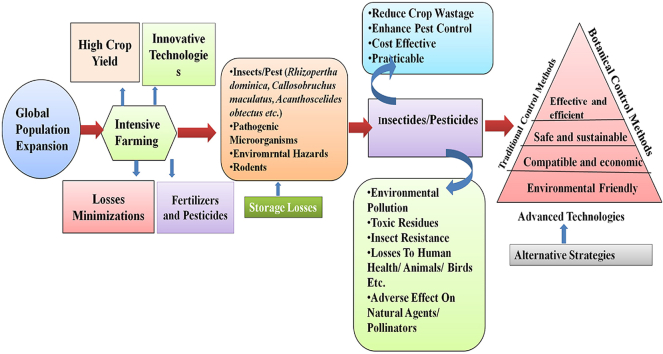

## 1 Introduction

Farming is the principal source of livelihood in the entire world. More than 70% population of Indian populationrelies on agricultural sector. Due to advancement in technology, our country has rapidly increased the production of pulses and grain in the past few decades. However, after post-harvesting period causes 10% loss in crops. Damage at the time of storage accounts for approximately 6% as appropriate storage services have not been available (Prakash et al., 2016). So, storage of grain facilities contributes important role to avoid damage caused by bruchid pests, disease-causing agents as well other animals. Various enemies such as rodents and insects attack the stored grains and infest them directly or indirectly. Hence, causes significant damages and other than storage losses.

## 2 *Callosobruchus maculatus*

**Figure 1:**
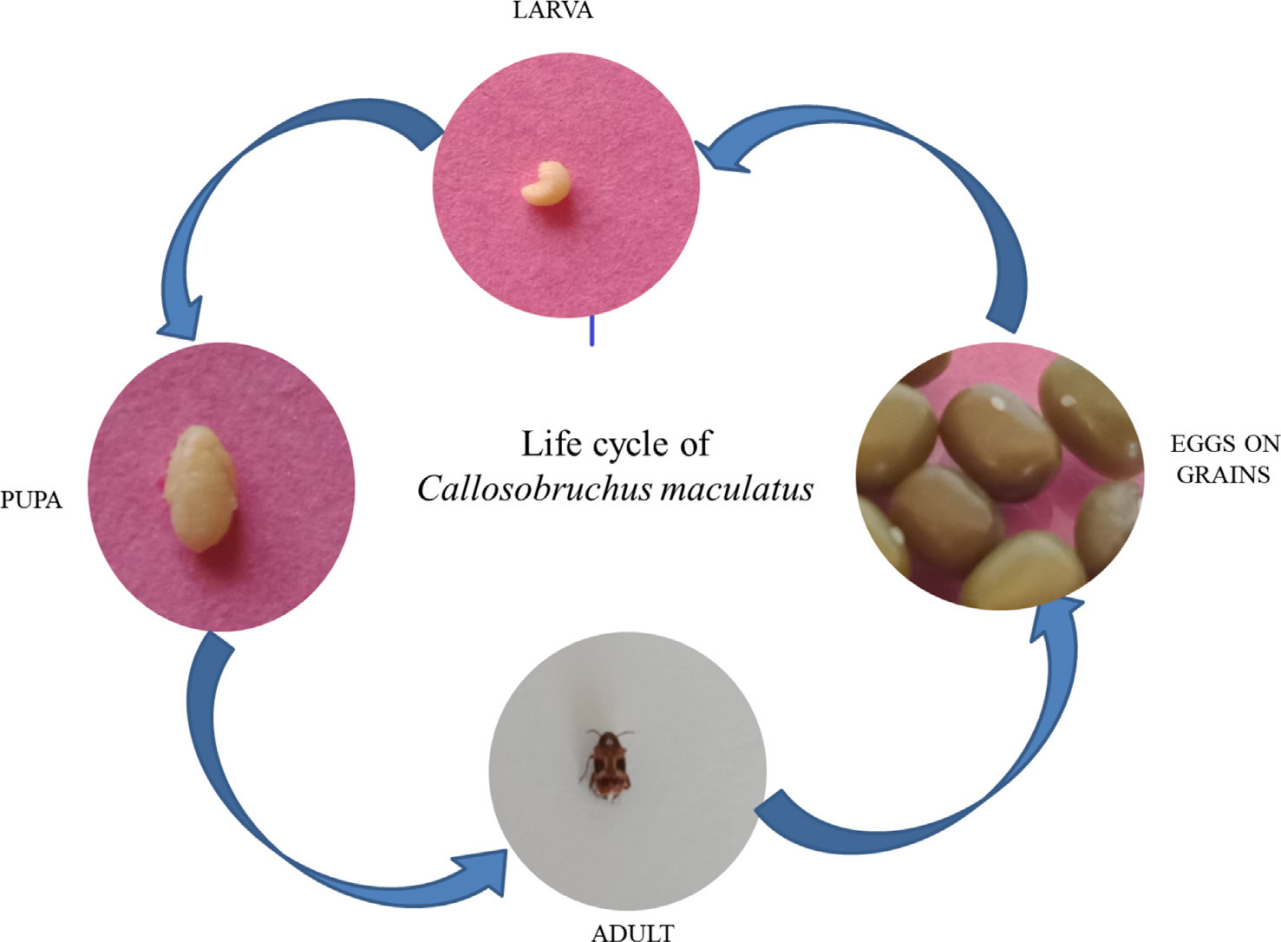
Life cycle of *Callosobruchus maculatus*.

Several pests mostly belong to order coleoptera (60%) and Lepidoptera (100%) (FAO, 2009; Atwal and Dhaliwal, 2008). Among all pulse beetles, *C. maculatus* causes severe damage to most pulses and a significant pest of Cowpea. Kergoat et al. (2008) taxonomically categorized this beetle in family Chrysomelidae and subfamily Bruchinae. Although several stored pulses are infested by this beetle and commonly recognized as cowpea weevil/bean beetle (Onyido et al., 2011). Females lay eggs on seed coat following the hatching first instar larvae enter into the seeds, developed by feeding on embryo grains and endosperm turned into pupae and complete its lifecycle by emerging as adult beetle (Figure 1). The first instar larva was characterized by a pair of pro-thoracic plates. These plates had capability to cut the hard seed coat and the larva penetrates into the seeds vertically from short distance (Augustine and Balikai, 2019).

The infested and damaged seeds become perforated and lead to loss of seed weight. Adult insects come out from through the emergence window following the completion of larval-pupal growth cycle. During the post-harvest storage, *C. maculatus* attacks the pulses and grains, causing significant loss one best example is cowpea on which *C. maculatus* becomes dominant (Park et al., 2003; Sanon et al., 2010; Adedire et al., 2011).

*C.maculatus* are the principal destructor of the pulses that account for 10–20% storage losses (Phillips and Throne, 2009). There are approximately 600 coleopteran species, which causes damage to grains in stores (Rajendran and Sriranjini, 2008). The insects causing damage to the stored grains often get access from the field and establish at the storage site due to micro-climate and retained during processing and storage (Hagstrum and Phillips, 2017). The initial infestation can be minimized during the postharvest handling of the storage structures by properly harvesting and drying the grains. These insect pests moved from one region to another through the commodity. Most of the problems spread in different places because of their flight habit (Mahroof et al., 2010). In India, crop damage has been estimated because insect pests range from 10-30% per year, out of which 26% is due to insect pests (Ridley et al., 2011).

Pulses infested by *C. maculatus* directly or indirectly, thus causing severe damage other than storage losses. Most of the storage part is mainly concentrating on grain storage either at a domestic or commercial scale. Several structures were used for storage of grains ranging from a small metal bin to tall grain elevators. Such stored commodities for a long-time cause contamination and damage by biotic and abiotic factors. Among the biotic agents, insects, mites, rodents, birds and microorganisms are causing colossal loss to store grains.

In addition to the harm by insect pests and diseases, there are insufficient and very poor storage facilities, causing enormous losses yearly. Various insect pests have modified themselves according to the diet of dried feeding material; some are important pests of grain that can make a hole in the healthy kernels. Initially, these insect pests make their path through the episperm of seed. It is estimated that all the activities of insect pests like feeding on grain, their presence in the cereal grains and products, and the expenditure for the strategies used to destroy them have caused a significant economic loss. If we can save these losses, our stockpiles of food grains could grow enormously, thus enabling us to feed millions of hungry people worldwide.

**Figure 2:**
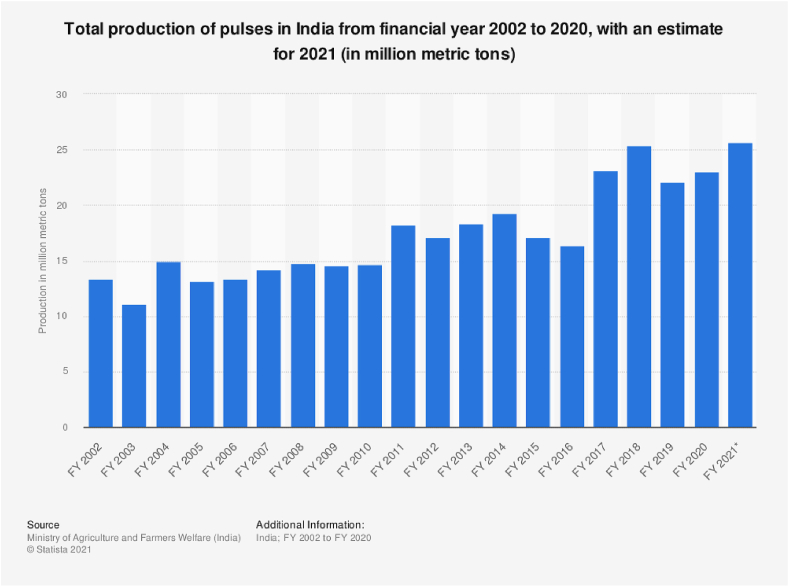
Financial Year (FY) wise India's pulse production during (2002–2021) (Source: Minister of Agriculture and Farmers Welfare (India), Statista, 2021).

The losses caused by insect pests and diseases are less apparent than those caused by erratic monsoons, fluctuations in global weather, improper storage and postharvest procedures. However, much could be done at various levels to reduce and possibly eliminate insect pests in the process of storage. In early 1960s, with the origin of Green Revolution such varieties having more productivity were introduced. It helped India to emerge as one of the leading developing countries, resulting in increased food grain production, touching more than 230 million tons per year (Anon, 2009). In India, there are approximately 22–25 million hectares of areas where pulse legumes are sown. There is overall increase in pulse production from the financial year (FY) 2002–2021 in South Asian Country (Figure 2).

## 3 Emerging risks of climate change

Despite surplus food production, there is still hunger and poverty, as million tons of valuable food grains amounting to more than 2000–3000 crores of rupees either gets damaged due to improper scientific methods of storage each year. Moreover, cereals and their processed byproducts are also prone to deterioration by diversifying biotic and abiotic factors. Together, these account for the loss of about 25% of food grains worldwide. These include heat, moisture content, relative humidity, microorganisms, insect pests and rodents. The climate alterations show its serious influence directly as well indirectly on farming, agriculture as well their related pests.

All these factors are directly associated with reproductive rate, development, fitness and migration of pest, while indirectly the global climate alterations influence the correlation between different pests, habitat, natural enemies as well their environment (Prakash et al., 2014). According to Fuhrer (2003); Kocmankova et al. (2009); Fand et al. (2012), temperature affects the various activities of insects such as life cycle, biology, growth as well reproduction because their body depends upon the surrounding temperature and environment. So, increased CO_2_, increased temperature in atmosphere also affects the survival of insect population as well crop losses percentages. Alterations in climate provide the new ecological niches which create chances for pests to grown up in changed habitats. This is the reason that farmers face the problem of new pests and year wise the problem get intense. The migration of these pulses and crop pests by crossing geographical boundaries becomes a global reason for food insecurity to various countries (FAO). Even, global warming also increases the population of pests, so increase in number of these pest species also disturbs the population balance (Menendez et al., 2007).

## 4 Importance of pulses

Pulses belonging to the Fabaceae family and are grown all over the globe for their proteinaceous seeds. Moreover, a rich protein source and human nutrition, these are also helpful for its fiber-rich diet, including carbohydrates, starch, vitamins, minerals etc. Product of these pulses could also utilize as low-valued animal feed and wood for the fire. Pulses also maintain the process of nitrogen fixation and increase the nitrogen and phosphorous content in the soil. These pulses could also enhance our living and nonliving surrounding (Patterson et al., 2009; Sardana et al., 2010).

Pulse grains are the main protein source in the developing society. Pulse proteins contain various essential amino acids (methionine, threonine, cysteine, tryptophan and lysine) So, these pulses provide important amino acids in combination with other minerals having high food value when complemented with other cereal grains. It provides the principal protein diet for vegetarians (Reddy, 2010; Saxena et al., 2010; Asif et al., 2013). Thus, pulses contribute a perfect mixture of crucial amino acids and other minerals having high biological value when complemented with cereals and considered the principal protein diet for vegetarian. According to dietary perspectives, pulses comprise a greater part of healthy as well as balanced diet and contribute the dominant portion of energy-rich substrate. Therefore, utilization of these pulses has increased during several decades. There was shortfall in pulses production compared to its demand by the world's growing population. Beetles belong to the family bruchidae have been closely related to legumes, and most of the species are the main pests of stored grains. The attack of bruchids is high in cowpea because of less natural resistance to insect in storage.

In addition to its deficiency of pulse production, the net productiveness and its economic value are perpetually affected by the storage pests, especially the bruchids. Pulse beetles are the bruchids beetle that initiates their attack in the fields, and sometimes during harvesting and cause 1–5% infestation. Most of the times, decreased population of bruchid can maintain developing larval and pupal population in the stored grain pulses during storage. Larval stage consumes the grains of cowpea and other pulses which affect the seed weight and its germination ability (Murdock et al., 2003; Deshpande et al., 2011). To save pulses from *Callosobruchus* spp. Infestation and achieve better results, there is a need for a systematic organization of the outcomes and a reframing of the research, which prove helpful and practical—the present review based on the potential of different control measures against *Callosobruchus*.

## 5 Methods

We searched for article published from year 2005–2021 in Google Scholar. Most of the articles related to bruchid (*Callosobruchus* spp.) and their control. Few old citations have to be mentioned wherever needed. We searched for article Science Direct, Elsevier and Springer for each management strategy: Cultural control, sealed containers, fine ash, intercropping, harvesting time, alternate host, effect of vegetable oil, heating and freezing effect, solar treatment, radiation treatments, different bacteria and fungus for microbial control, natural parasitoids and predators, botanical extracts, chemicals, resistance varieties of pulses and also their efficacy for pest management. The latest article related to biotechnological techniques and cold plasma treatments were also searched Nature Portfolio, and data bases used (SCOPUS and WoS). We excluded study regarding other grains, agriculture and horticulture field pests. Integrated pest management was also focused through the search of recent innovations and applicability.

## 6 Results

**Figure 3:**
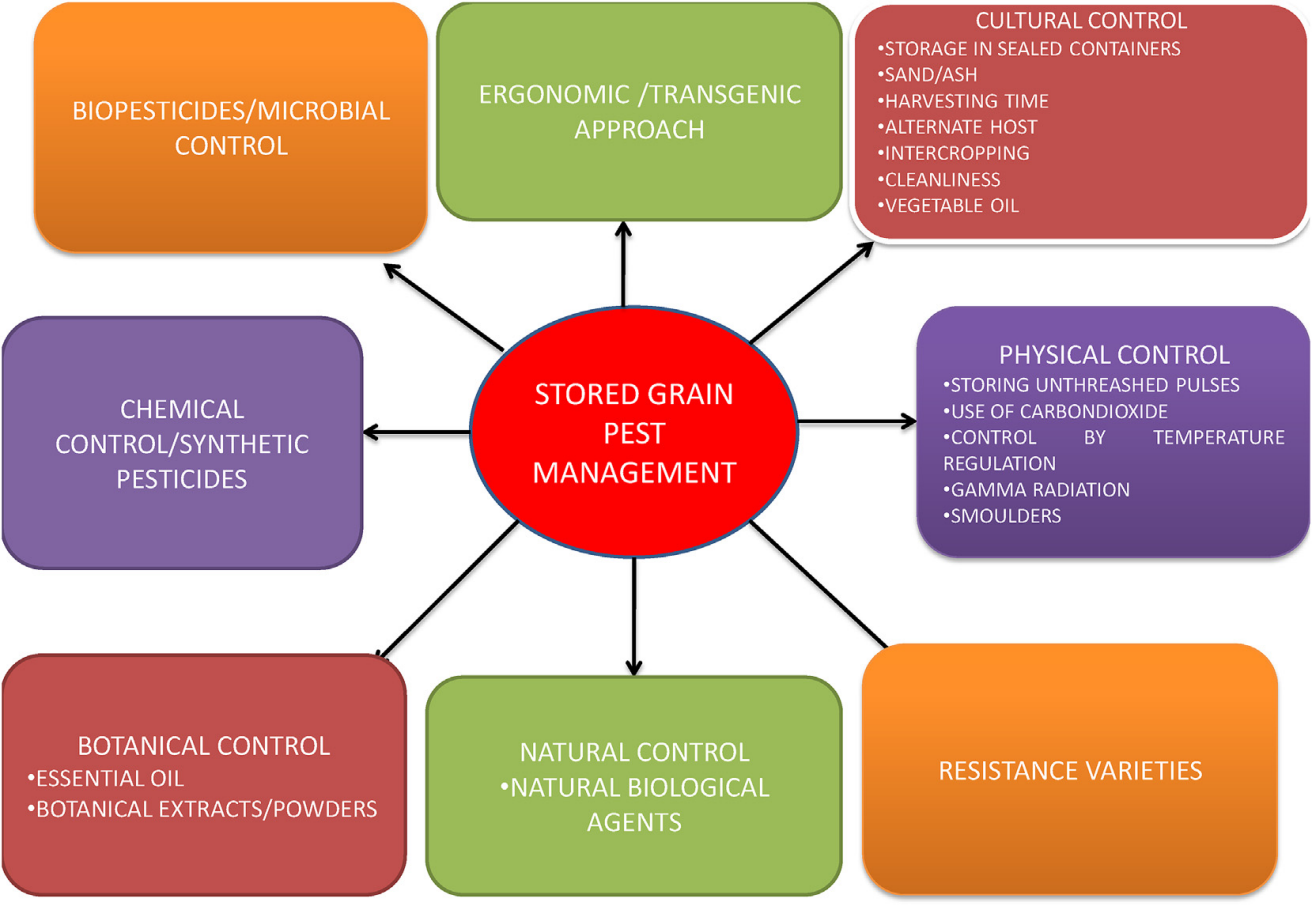
Pest management practices for stored grain pests.

In the present time, bruchids cause enormous loss to pulses and a severe issue of concern. Several strategies have been developing for the prevention of vibrations from insect infestation, especially *Callosobruchus maculatus*. There are many methods and control measures taken against storage beetles are (Figure 3).

### 6.1 Traditional pest management approaches

The host environment is manipulated in traditional cultural control, which eradicates the prevalence of stored grain pests. It includes sealed containers, ashes, managing harvesting time, and discarding infested grains before storing fresh grains. Fumigation, painting and white-washing the walls of storehouses act as an insect repellent. These strategies are less attractive, but suitable to control developing bruchid. All the traditional practices are shown in Table 1.

**Table 1:** Traditional pest management approaches.

Sr no.	Strategies	Materials and Methods	Mode of action	Stage affected	References
1.1	Sealed containers	Plastic containers/polythenee bags/Iron containers	Asphyxiation	Adult	Shukla et al., 1993; Seck et al., 1996
1.2	Inert Materials	Sand/Ash/diatomaceous earth/silica aerogel/non-silica dust	Asphyxiation	Egg, larva adult	Chinwada and Giga, 1997; Subramanyam and Hagstrum, 1995; Quarles, 1992
1.3	Harvesting time	No previous infestation	Absence of adult pest	-	Caswell, 1968
1.4	Alternate host	Eliminating wild host plants	Prevent continued infestation	Egg, larva	Pimbert (1985)
1.5	Inter-cropping	Non host plant	Lethal to developing stages	Egg, larva	Alzouma, 1987
1.6	Storing Un-threshed Pulses	Pod wall	Barriers for egg development	egg	Throne et al. (1990)
1.7	Cleanliness	Use of DDT and BHC and other chemicals in Old sheds, godowns	Removes previous infestation by Asphysiation	Egg, larva, pupa, adult	Caswell, 1976
1.8	Smoulders	Smoke	Fumigation	Adult	Keever and Cline (1983); Gilbert (1984)
1.9	Gaseous Effect	O_2_ and CO_2_	Fumigation	Eggs and adults	New and Rees, 1988; Mbata and Reichmuth, 1996
1.10	Vegetable oil	Crude and non edible oil	Ovicidal property	Egg	Schoonhoven, 1978; Naik and Dumbre, 1985

#### 6.1.1 Storage in sealed containers

Storage beetles can enter into plastics up to 0.18mm thickness. Therefore, in various regions of world, to store grains, plastic bags with a cotton lining and the jute bags are commonly used for packing. It has average cost as well better capacity to resist the bruchid pets. Moreover, it can be used several times because of its good quality and also the less risk of the tearing. Hermetically sealed triple-layer plastic bags were also used in some countries like Niger (Baoua et al., 2012a, b). In these bags, beetles will consume all the existing oxygen immediately and thus choked. So, their development suffers However, even a minute hole in the storage bag will show negative results because air can enter the bag. Storage bags should also be protected from rodents, while iron containers should be prevented from rust and treated with care to prevent destruction from pest. In various countries like Brazil proper airtight storage containers were used for storing cowpea seeds which protect them against *C. maculatus* and *C. analis.* Polythene sacs were also used for grain storage and also important because it asphyxiates the pest. It is a simple and more effective physical method in which altered atmosphere were provided to several developing instars of bruchids(O_2_< 1%, CO_2_ = 9–10%). 100% mortality was obtained within two days for adults. At the same time, other developmental stages such as egg, larva and pupa were also affected (Mohapatra et al., 2014).

#### 6.1.2 Sand/ash

An inert material such as sand and ash are mixed with stored beans, preventing them from stored beetles. This inhibits the sexually mature insects to find the mate and copulate as well laying eggs on seeds. Fine ash or powders, can be effectively asphyxiating the adults, and also lethal to egg or larval stages inside the seed (Bamaiyi et al., 2007; Tabu et al., 2012) Sometimes, it also inhibit 100 % adult emergence (Karimzadeh et al., 2020). Paddy husk ash, could be also used against *Callosobruchus maculatus* (Ashamo et al., 2021; Atewoja et al., 2021). This method of seed protection is practically less used because a large amount of inert material is required, particularly during the large number of grains. Conventionally, various sand and soil components are used as inert dust. In some cases, products of diatomaceous earth were used and applied with different doses in lab conditions and found effective against various bruchids (such as *C. maculatus, A. obtectus*) infesting cowpea, kidney beans etc. It also causes high adult mortality (Stathers et al., 2004).

#### 6.1.3 Harvesting time

The harvesting time of cowpea and another pulse should be managed, which results in the decline of 50–90% of damage alone done by Cowpea beetle. The pulse crop should stay behind in the field until it is completely matured and developed. Adults choose to detain their attack to the uncovered seeds. Pre- and post-harvest losses were also found due to *C. maculatus*. Immediate harvesting of fully grown pulse pods would decrease the infestation of *C. maculatus* and also damage to grains. So, it should be ensured that during harvesting there was low pest population. Significantly, more damage was noted for the seeds during the harvest after 80 days than the 60 and 70 days (Baidoo et al., 2010).

#### 6.1.4 Alternate host

Sometimes, the alternate plant which can be host for the particular pest grown side wise. It meets the requirements of pest when actual pulse grains plants were not available. The pest survives and also completes generations sidewise. In some regions of Costa Rica, *Phaseolus lunatus* was the host plant of pest *Zabrotus* *subfasciatus* in wild conditions (Pimbert, 1985). They maintained their life cycle on *Phaseolus lunatus* wild plant. So, infestations can be reduced by eliminating such kind of plants from cultivated land.

#### 6.1.5 Intercropping

Growing two compatible crops at one time is known as intercropping. It was also found that bruchid (*Acanthoscelides* *obtectus*) infestation of bean can be minimized with the help of maize crop. In Nigeria, the intercropping system were incorporated and found that it decreases the cowpea infestation by bruchid pests during pre and post-harvest storage. During experiment four different and susceptible pulse varieties of cowpea (Borno brown, Kanannado, IT93-637-l and 1T89KD-374-5) were taken. These were grown with pearl millet both as intercropping system and singly without pearl millet. This intercropping help in reducing *Callosobruchus* infestation and high crop yield (Kabeh and Lale, 2008). Earlier, when cowpea sown with millet, there has been negligible effect of bruchids beetle (*Bruchidius* *atrolineatus* (Alzouma, 1987).

**Storing unthreshed pulses:** during the storage of unthreshed pulses the bundles of pulses plants were tied with plastic rope, sometimes ropes made up of other plant branches. Then these bundles were stored on the tree or other safest place (Ofor, 2011). The pulse grainsremain in the open air and become fully dried. Farmers used them when they need for utilizations and commercialization. In various developing countries the most of cereal grains (threashed/unthreashed) are reserved at home with the traditional methods (Nduku et al., 2013).

Un-threshed grain means to which stalks or other parts of the parent body are not removed. Van Huis, 1991 found un-threshed pulses or seeds inside the pods comparatively decreased infestation. Pod walls act as a barrier against insect pest infestation. If the pod wall of grain/seed is thick, there would be minor infestation and high larval mortality. The author proved that the first instars larva gets physically exhausted during the cutting and entry through the pod wall. The pods should be attached for a long time with the plants because unbroken pods provide better protection against pests. This practice is compatible with IPM and provides a better alternate strategy to suppress bruchid population as well maintain seed quality. Although there are some limitations such as screening of specific proteins in the seeds that are toxic to bruchids as well identification of resistant germplasm (Throne et al., 1990).

#### 6.1.6 Cleanliness

Cleanliness is most important during the pulse grain delivery from field to warehouses. Old sheds for storage, carriage vehicle should be cleaned; probably it eliminates infestation to some extent. The insect infestation rates are high in the tropics. So, there is a need for a good and clean grain storage environment. After harvesting, grains have been protected in granaries, decreasing the pest infestation. Proper sun drying and repeated sieving of pulses could also prevent bruchid infestation (Mohapatra et al., 2014). If the bruchids present in storehouses move towards the adjacent field and cause an infestation in the field also. Therefore, damaged or egg-bearing seeds should be properly disposed of from the granaries.

#### 6.1.7 Smoulder

Cow dung cakes and various plants produce smoke. It was found fatal to insect pests without showing any side effects on germination as well post germination of pulse seeds. This is being easily available, cost effective, biodegradable, un-harmful and simple to proceed by small holders. It can be an alternate for chemical insecticides as well fumigants for storing grain (Yadav and Tiwari, 2018). Cow dung smoke even be more effective to control pulse beetle and also cause 50% mortality within 96–120 h (Kishor and Tiwari, 2021).

In Africa, cowpea seeds are occasionally stored for a short time above the kitchen fire in homes. It is advantageous because the developmental growth of the pest is maintained and also preventing re-infestation. High temperature, as well as smoke, also has repellent action. Gilbert (1984) also demonstrated that blacklight traps could be more helpful in controlling and trapping *C. maculatus*, especially females.

#### 6.1.8 Gaseous effect

The oxygen is sole responsible for respiration to all organism including insects. If anytime concentration of carbon dioxide extremely increased in storing conditions. This makes the atmosphere unsuitable and also acts as poison to insects (Navarro, 2012). The high carbon dioxide and low oxygen levels kill adult beetles and oxygen-free environment or 100% carbon dioxide is more toxic to developing stages of pests. Overall mortality of insects was found with increased carbon dioxide levels. It should be used for favorable time and ensure that most of insects must die. The most affected stage was egg and early developing stages. Even 18% carbon dioxide concentration was helpful in insect pest (*C. maculatus*, *A. obtectus* and *Z. subfasciatus* etc.) mortality (Cheng et al., 2013; Wong-Corral et al., 2013). When oxygen, carbon dioxide and nitrogen, the three superior gases were used against *C. maculatus* in mixed combination then it found effective because of the surroundings were altered. More efficient results were found with reduced nitrogen and enhanced carbon dioxide level. This can eliminate synthetic insecticides and provide chemical free food (Ingabire et al., 2021). Recently some authors also used ozone gas against *C. maculatus* and *C. chinensis* (Gad et al., 2021).

#### 6.1.9 Vegetable oils

In some parts of Asia, vegetable oil (Approximately 1–15 ml per kg seed) against insect pests is a widespread practice. Various authors have verified the effectiveness of oil against *C. maculatus; C. chinensis* and *Zabrotes* *subfasciatus.* Full-time oil protects pulses from pest attack. Crude and non-edible oils gave better protection to seed grain in comparison to purified and non-edible oils. These oil coatings have ovicidal property. It causes poor oviposition and inhibits the egg laying and high larval and adult mortality without affecting germination and cooking quality of seed (Boeke et al., 2004; Wanderley et al., 2020). Sometimes different edible and non-edible oils were also used against insect pests (*C. maculatus*) to prevent its infestation. The oils at different concentrations were also used to check their efficacy on egg laying and adult longevity (Ake, 2011).

These cultural practices are considered the cheapest, easy to use methods, for small holders, during pre- and post-harvest time. However, these methods are less used, because of several disadvantages like, need for preterm planning and appropriate and proper timing as well species-specific methods (Kananji, 2007; Gupta et al., 2016).

## 7 Modern pest management approaches (Table 2)

### 7.1 Physical control/control by temperature, freezing and heating

This method primarily depends on the management of the grains and insect pests with the help of physical agents, such as temperature, heating, humidity and pressure (Sahadia and Aziz, 2011). Every insect species has a suitable environment, including ambient temperature, relative humidity, and photoperiod, which help its growth, proper development, and high reproduction. Therefore, alternation to the temperature and relative humidity from its optimum period, then the developmental growth of insect pests can be reduced and stopped. In addition, the different developmental stages such as egg, larva and pupa of various *Callosobruchus* spp. have been killed by allowing the pulse grain to remain in the temperature up-to 60–65 °C for a few minutes or reducing the temperature to below 12 °C (Sahadia and Aziz, 2011). Upadhyay and Ahmad (2011) studied the two pulse seeds (cowpea and moth bean) and found that the lowering of humidity below 9% can also prevent the fecundity and developmental growth. Eggs and larvae of *C. maculatus* can also be killed by lowering the pressure and increasing temperature. In some cases, the solar heating method has also been a good option against bruchid beetles infesting green gram and cowpea and other pulses. It can make pulses 100% free from infestations (Chauhan and Ghaffar, 2002; Mbata et al., 2005; Moumouni et al., 2014). The temperature of seeds can be increased to 52 °Cto 65 °C, when these were kept on black polythene sheet under the sun. This method causes the death eggs and developing stages of *Callosobruchus* effectively. Disinfestation unit can be driven with the heat of solar energy (Gbaye et al., 2011; Baoua et al., 2012a, b; Ajayi et al., 2021). The treatment with solar heat was found that the pest outbreak become negligible during storage conditions. It also increased germination capability of seeds (Maina and Lale, 2004; Fawki et al., 2014). This treatment can also be used for various stored grain pests (Hansen et al., 2011).

**Table 2:** Modern pest management approaches.

Sr no.	Strategies	Materials and methods	Mode of action	Stage affected	References
2.1	Physical Control	Temperature, freezing and Heating	Inhibit developmental growth	Egg, larva, pupa, adults	Sahadia and Aziz, 2011; Moumouni et al. (2014)
2.2	Radiation Treatments	Beeta and Gamma radiatins	Inhibit developmental growth	Egg, larva, pupa, adults	Valizadegan et al. (2009); Fawki et al. (2014)
2.3	Resistance Varieties	Resistant grain wall	Inhibit developmental growth	egg	Miesho et al. (2018); Swamy et al. (2019).
2.4	Natural control	Biological agents, parasitoids	Lethal to developmental stages	Egg, larva, pupa	Eliopoulos, (2006); Cortesero et al. (1997); Kananji, (2007).
2.5	Phyto-chemical control	Botanical extracts/powders/Nanopesticides	Repellent, deterrent and lethal action	Egg, larva, pupa, adult	Elumalau et al. (2010); Arora et al. (2017); Elango et al., 2016; Andy and Edema, 2019
2.6	Chemical control	carbamates, organophosphates, organochlorines, and pyrethroids.	Lethal action	Developing stages and adults	Talukder (2009); Parmar and Patel (2016)
2.7	Microbial control	Microorganism	Lethal Action	Developing stages	Phillips and Throne (2009); Kavallieratos et al. (2012a), b; Gahukar (2014)
2.8	Transgenic approach	Alteration of DNA, Genetic maping	arcelins, phyto-hemagglutinins and *α*-amylase as bruchid inhibitors	Larva	Collins et al. (2006); Liu et al. (2006); Qaim (2010); Rasoolizadeh et al. (2016)
2.9	Cold Plasma Treatments	high-voltage air-based atmospheric cold plasma	Reduces respiration rate and cause asphyxiation and lethal	Egg, Larva, pupa and Adults	Ziuzina et al. (2021); Pathan et al., 2021

### 7.2 Radiation treatments

Gamma radiations are lethal to adult stored grain pests, their eggs, developing larvae, while adult females of pests are more sensitive to this treatment. Gamma radiations can cause complete mortality of insect pests of stored grains. Ionizing radiation produces high energy particles which break the chemical bonds and cause DNA fragmentation of pest in unusual way (Todoriki et al., 2006; Hasan et al., 2012). Authors also used a metal box heater with gamma radiations. This metal box causes complete (100%) mortality of beetle in few minutes (Fawki et al., 2014).

The author also states that *β*-radiation is easy to handle and safe; as its operating system is easy, farmers can easily apply it, while, *γ*-radiation are isotope-based and applied continuously, which is unsafe for humankind. Microwave radiations are also helpful with low-temperature treatment. With this strategy, pest management such as *Oryza Theophilus surinamensis* was done in wheat (Ghasemzadeh et al., 2011). Valizadegan et al. (2009) found these treatments as effective and eco-friendly in pest management Programmes. Besides their successful aspects, these irradiation management techniques of temperature regulations have some limitations, such as high price, radiation system handling and germination inability of seed (Mishra et al., 2018).

### 7.3 Resistance varieties

*C. maculatus* infests the cowpea grains, but various cowpea varieties show resistance against this pest. This is because of pod wall, grain covering texture and protein content, of resistance cowpea varieties. All resistant pulse grains are not equally vulnerable to the attack of *C. maculatus*. Even there would be a different developmental period for wild and cultivated cowpea variety (Ogunkanmi et al., 2013; Badii et al., 2013). Ileke et al. (2013) studied 31 cowpea varieties and found that these varieties are susceptible to *C. maculatus. Phaseolus vulgaris* also show high resistance against *Zabrotes* *subfasciatus and Acanthoscelides* *obtactus.* The seed materials, hardness and chemical property of the seed act as an obstacle for insect pests and were found fatal to the larval stages of *C. maculatus*. The cowpea variety (TVu, 2027) has high resistance against *C. maculatus*. It contains trypsin inhibitor in high concentration. It also possesses amino acids having Sulphur which acts as an antibiotic to developing larvae. So there is less survival rate and prolonged development. Dick and Credland (1986) reported that grain beetles rapidly improve their developmental growth against different varieties of pulses. The seed coat of chickpea was found resistant to *Callsobruchus maculatus* because this inhibits Oviposition. Most of the time, *Callsobruchus maculatus* choose smooth-walled seeds, whereas wrinkled seeds are avoided (Messina and Renwick, 1985). (Dharmasena and Subasinghe (1986) evaluated 12 varieties of green gram and found them resistant to *Callosobruchus* spp. It is most effective, efficient, easy to use, cost effective methods for the control of stored grain pests and can be easily adopted by farmers. It requires screening of various seeds having such strains which are resistance to bruchid pests. There are various varieties of pulse seeds (chick pea, green gram, black gram etc.)which shows resistance against *Callosobruchus* (Swamy et al., 2019; Kaewwongwal et al., 2020). Various other authors also evaluated the resistance cultivars of pulses which is resistance to the infestation of *C. maculatus*. This resistance of cutivars related to larval ability of bruchid pests (Amusa et al., 2014; Lopes et al., 2018; Miesho et al., 2018; Messina et al., 2019).

### 7.4 Natural control strategies

It involves living organisms, which are identified asbio controls agents (predators, parasitoids, pathogens etc.). These are used to retain the insect pest populations below the destruction level, and most of the time; no loss will occur (Altieri et al., 2005; Mahr et al., 2008). It is regarded as a practical or satisfactory technique where important natural agents have been used against stored pests. These natural enemies effectively control the insect pest population. These mainly include the egg, larval and pupal parasitoids. Egg parasitoids lay their eggs on the eggs of developing bruchids beetles, for example, *Uscana* *lariophaga*. While larval and pupal parasitoids lay their eggs on developing larva and pupa such as *Dinarmus basalis* and *Eupelmus* *vuilleti*. This strategy can control bruchids infestation up to 82% under optimized laboratory conditions (Cortesero et al., 1997). These parasitoids may suppress beetle populations in the field, but not up to 100% (Gahukar, 1994). Soundararajan et al. (2012) reported that a hymenopteran parasitoid (*Dinaramus spp.*) had been used against *C. maculatus* population infesting urdu bean. Similarly, *Bruchus Chinensis* (bean weevil pest) has been controlled by a parasitoid (*Apanteles* *flavipes*) (Eliopoulos, 2006). Various authors Schmale et al. (2003), 2006; Campan and Benrey (2004); Sood and Pajni (2006); Velten et al. (2007), 2008 provide the details of natural control agents for bruchids. Such as *Dinarmus basalis*, *Stenocorsebruchivora* used to prevent infestation of *C. maculatus* and *Pteromalus* *cerealella* respectively Schmale et al. (2003); Velten et al. (2007), 2008; Sanon et al. (2002); Campan and Benrey (2004). This biological control method has some limitations when used by small scale farmers. Peoples don't know to maintain the parasitoid culture, nutrition to parasitoid larvae and timing to release the parasitoid (Kananji, 2007).

### 7.5 Phytochemical/botanical control

**S**econdary metabolites (such as alkaloids, phenolics, and terpenoids)in plants which possess the property that affect the sense organs of plant-feeding insects, preventing them from egg-laying and feeding (Ake, 2011). Huang et al., (1997) demonstrated and found that nutmeg (*M. myristica*) oil has antifeedant property against *Sitophilous* *zeamais* and *Tribolium* *castaneum*. Various medicinal plants contain different essential oils that were used against stored product insects (Elumalau et al., 2010). Andy and Edema (2019) evaluated that the five plant materials, main spices such as African nutmeg, Manjack, Ginger, Galic, and Negro Pepper, were effective against cowpea weevil. This resulted that Ginger extract inhibits feeding in *C. maculatus.* Fawkes et al., in 2014 found the potential of lemon and orange peel powder on *Callosobruchus* *macualtus* and lemon peel powder shows more effectiveness. Arora et al., 2017 found various hurdles for providing these botanical extracts to small scale farmers. Such as the non-availability of plant's extracts at the commercial level. So, farmers are unable to replace chemically synthetic pesticides. Moreover, the production and distribution problem of plant-based pesticides are the main challenges. Therefore, there is a need to start an awareness campaign that discusses the use of plant-based insecticides with local people and farmers (Grzywacz et al., 2014).

Several studies found that *Piper nigrum* had better insecticidal potential, which controls bruchids infestation and protects stored cowpea seeds. Various authors agreed with the results that members of the Piperaceae family possess various bioactive agents such as piperine and chavicine, which show high insecticidal activity against various crop pests (Lale, 2002; Okonkwo and Okoye, 1996; Adedire and Lajide, 1999). Although, powder extract of *Afromomum* *melegueta* did not affect fecundity, egg-laying capacity as well as egg hatchability of *C. maculatus* (Ofuya, 1990). Onekutu et al. (2015) studied and found that if any plant extracts did not control *C. maculatus* within 24 h, a particular concentration is not considered suitable botanical extracts. Contact and fumigation are the main modes of action in plant material extracts (Asawalam and Emosairue, 2006; Asawalam et al., 2007; Franccedil et al., 2009; Ukeh et al., 2010). Manju et al. (2019) also evaluated the efficacy of twelve botanicals (*Ipomea* sp., *Ocimum sanctum*, *Pongamia pinnata*, *Vitex negundo*, *Adhatoda*sp., *Zingiber officinale*, *Allium sativum*, *Curcuma longa*, *Acorus calamus*, *Capsicum annum*, *Piper nigrum* and neem seed kernel powder were found effective to control bruchid pest, *C. maculatus* in green gram seed storage. The essential oil of *Chenopodium ambrosioides*were used against *C. maculatus* and shows high toxic as well repellent effect on it. It also affects mortality rate, fecundity and reproductive capacity. Most of the plants are edible and nonhazardous unlike other synthetic pesticides (Elhourri et al., 2021).

Raja and Ignacimuthu (2000) found the efficacy of groundnut or coconut oil against *C*. *maculatus* and have a toxic effect on fecundity and development. Tobacco plant powder extract is found to be more effective in hindering fecundity and hatching of eggs of *C. maculatus* infesting cowpea. The leaves, stem, roots and flowers of *Moringa oleifera* had been considered botanicals against bruchids. This was studied under ambient temperature conditions (30^∘^C) and humidity (72%) in the Biological Oxygen Demand Incubator. These extracts reduce fecundity, hatching and adult emergence (Ofuya and Akhidue, 2005; Adenekan et al., 2013). Idoko and Ileke (2020) studied insecticidal property of five botanical seed oil used against *C. maculatus* and essential oil from *Aframomum* *melegueta* was found most effective. Various nanoparticles were synthesized from the extracts of different plant parts (bark, leaves, flowers, stems, roots etc.). Nano-particles prepared from *Alternanthera dentate* leaf extracts, pine, persimmon, ginkgo, magnolia and platanus, *Annona squamosa, Coriandrum sativum*, *Cocos nucifera*, *Scadoxus* *multiflorus*, *Pongamia pinnata* and many others (Kumar et al., 2014; Song and Kim, 2009; Senthamilselvi et al., 2013; Sathyavathi et al., 2010; Elango et al., 2016; Malaikozhundan and Vinodhini, 2018).

Harshani and Karunaratne (2021) studied the efficacy of citrus fruit peel powder against *C. maculatus* and also found effective. Kosini et al. (2021) evaluated the insecticidal property of *Gnidia* *kraussiana* extracts. It also showed toxicity on egg and larval stages of *C. maculatus*. Various plant leaf, seed, bark, root powders as well their fumigant activity are used in various countries to inhibit seed/grain damage and have negligible effect on human health (Kestenholz et al., 2007; Lehman et al., 2007; Mario et al., 2021). To prevent grains from pests the powder form of plant parts can be directly incorporated into grain bag and also as fumigants. It can cause death of developing stages of pests (Shaaya and Kostyukovsky, 2006; Sadeghi et al., 2006; Rajendran and Sriranjini 2008; Kumar et al., 2009; Shimizu and Hori 2009; Coelho et al., 2010). Radha and Susheela (2014) also formulated a product of neem seeds and curry leaves which was found too effective and decline the bruchid pest population in stored cowpea. During the botanical extracts study till date, we did not find any side effects for human as well animal.

### 7.6 Chemical control/synthetic pesticides

Four groups of pesticides come under chemically synthesized pesticides, such as carbamates, malthion, organophosphates, organochlorines etc. Mainly these pesticides were used against bruchids and other storage pests Dent, 1991; Megerssa (2010). Harberd (2004) had also demonstrated the efficacy of synthetic pesticides. It includes fumigants, dust and sprays for the prevention of bruchids. Synthetic pesticides, such as Acephate, Propoxur, Metaldehyde, Boric Acid, Diazinon, Dursban, Malathion, permethrin, lindane, phostoxin, methyl bromide and iodofenphos etc. when used at large amount then it causes the addition of toxic component on treated products. However, the bruchids pests also make resistant against these synthetic pesticides (Talukder, 2009). Parmar and Patel, 2016 evaluated the effectiveness of nine synthetic pesticides against *Callosobruchus*. Synthetic pesticides should be used in the correct amount and with accurate application methods. Then it produces effective results. Most of the families in villages don't have such knowledge, and it creates health problems and sometimes fatal to consumers. Other disadvantages of this method are that it kills other beneficial insects and negatively affects seed germination (Ramzan, 1994). So, there is a need for alternative approaches for bruchids management, such as using biotechnological tools and producing hybrid plants and production of biopesticides.

### 7.7 Biopesticides/microbial control

*Beauveria bassiana* and *Metarhizium* *anisopliae* and were used as biopesticides against *C. maculatus* which inhibit the population growth of pest (Cherry et al., 2005). Phillips and Throne (2009) studied the biotic and abiotic environment of pest and found that the alternation in pest environment could be effective for its control. Microbial control includes the use of microorganisms (bacteria, fungus, viruses etc.), pheromones, growth regulators etc (Kavallieratos et al., 2012a). Natural predators are also considered in this strategy. This is also an environmentally friendly, effective and relatively simple technique. Besides its advantages, this strategy there is less expenditure in microbial research; defind space; less infrastructure, as well few awareness campaign in developing nations (Gahukar, 2014). *Trichoderma harzianum* and various strains of entomopathogenic fungi also possess insecticidal potential against *C. maculatus* and *C. chinensis* (Abdelgaleil et al., 2021; Kordali et al., 2021).

### 7.8 Ergonomics approach/transgenic approach

The alternation of DNA with the help of recombinant technology comes under an ergonomic approach. It is an emerging technology that includes transgene introgressive, genome modification, DNA marker-assisted breeding etc (Collard and Mackill, 2008). Transgenic approaches have in vitro propagation tools as well as advancements in genetic engineering. Popelka et al. (2006) reported the use of DNA alternation in *Vigna unguiculata*. Earlier it was studied in *Vigna mungo, Cicer arietinum Cajanus cajan, Vicia faba* and *Pisum sativum* (Saini et al., 2003; Geetha et al., 1999). Biochemical research found that the pulses have specific proteins that generally present in the seed and contain plant defensive properties against bruchids. The most representative components against bruchids are various inhibitors (such as arcelins, phyto-hemagglutinins and *α*-amylase) (Peumans and Van Damme, 1995; Lioi et al., 2003).

Ryan (1990) studied that when lectins, *α*-amylase and protease inhibitors were taken with diet by the bruchid pest, it affected the larval midgut and retard growth and development of the pest. These findings were also supported at later stage by other authors (Ishimoto and Chrispeels, 1996; Ussuf et al., 2001). This is a safe option because these *α*-amylase inhibitors are easily destroyed during food preparation. So, with this technique GM crops can be developed having bruchids resistant properties. Various bruchids resistant transgenic pulses were developed, such as azuki bean, pea, mung bean etc (Sonia et al., 2007; Rasoolizadeh et al., 2016). This type of transgenesis is an effective strategy and has disadvantages, such as insecticidal proteins may affect even non-target organisms. Some studies found that when these trans-genetically modified peas and other grains fed by rats, chickens, etc., it also shows side effect on the development of those organisms (Collins et al., 2006; Liu et al., 2006; Qaim, 2010). So, the application of such crops is controversial that transgenic pulse crop is suitable for human or animal utilization.

Some varieties of cowpea have recombinant inbred lines which are multi multi-parent and advanced-generation intercross. Scientists identify such type of variants which possess resistant against seed beetles. The gene-to-gene interaction concept was also associated with resistance. Recently specific type of protein (arcelin) was identified which were present in wild bean and shows insecticidal actions against bruchids and affect their metabolism. The alterations in their own enzymes against the arcelin shows defence mechanism of insects (Messina et al., 2021; Hilda et al., 2021).

### 7.9 Cold plasma treatments

Cold plasma methods mostly used for food preservations by killing ffod bacteria and freezing temperature. It also has potential to control stored grain pests. Resistance is common problem in insects that can be developed against chemically synthesized pests. Cold plasma treatments were studied with chick pea seeds for about four years. During this study it was found that chickpea seeds were perfectly resistance against *Callosobruchus* sp. It also be used to inhibit various stored grain pests and also supports sustainable stored grain pest control. Further studies were also needed regarding to this concept and also to develop such machinery which can be used at commercial level (Pathan et al., 2021). Various problems related to control of insect pests can be solved with the cold plasma treatments and can also be a future alternative for protection of stored seeds. The seeds could be stored for longer periods. In the mechanisms of this high-voltage and air-based atmospheric cold plasma machine used a dielectric barrier discharge reactor and also were investigated against pests. Cold plasma treatment decreased both the respiration and the weight of insects and produce oxidative stress in adult bruchids (Ziuzina et al., 2021).

## 8 Integrated pest management (IPM) and their usage

The most important strategies and methods are those which can be operative. Even this can be applied for large as well small holders. It is the management strategy which incorporates the old as well modern strategies. The IPM in whole a management system which integrates the pest control measures (Shaaya and Kostyukovysky, 2006). This mainly focuses on such practicable methods which reduce the pest population in environment friendly and at low cost. Most suitable strategies were accepted and that can be either simple or complex but should be according to the IPM system. IPM is widely used to control pests especially of stored grain pests (Kavallieratos et al., 2017; Hagstrum and Phillips, 2017). It provides the knowledge about the different methods, that at what time these were incorporated for bruchid and other stored grain pest control. It can be improved by making better decisions. Many industrial applications can also influence the progress of pest management program in raw and also advanced as well processed commodities ((Hagstrum et al., 1999; Trematerra and Fleurat-Lessard, 2015). They are important during the designing of a pest management program. It also encourages new method to incorporate existing methods for the betterment of pest management (Hagstrum and Athanassiou, 2019).

## 9 Future perspectives

Since time immemorial, bruchid pest management is essential. Stored grain pulses were protected for many years. Various synthetic insecticides were incorporated with several bruchid pests of stored seeds, but considerable these were hazardous. Some practices have some limitations affecting non-target organisms, resistance, pollution etc. While these practices discussed above are essential in one and another way. But use of plant botanicals in single and combination may also be recommended. They are low cost, high availability, environment friendly and safe to human health during the control of *C. maculatus* as well other stored grain pests. These are significant in protecting pulses from insect invasion at farmer level. In another way there should be the combined use of different practices, inexpensive traditional strategies or advance Botanical, ergonomic and microbial practices and a combined integrated pest management approach.

## Declarations

### Author contribution statement

All authors listed have significantly contributed to the development and the writing of this article.

### Funding statement

This research did not receive any specific grant from funding agencies in the public, commercial, or not-for-profit sectors.

### Data availability statement

No data was used for the research described in the article.

### Declaration of interests statement

The authors declare no conflict of interest.

### Additional information

No additional information is available for this paper.
